# The Northern Finland Birth Cohort Eye Study: Design and baseline characteristics

**DOI:** 10.1186/1471-2415-13-51

**Published:** 2013-10-08

**Authors:** Ville Saarela, Elina Karvonen, Katri Stoor, Pasi Hägg, Marja Luodonpää, Jaana Kuoppala, Anja Taanila, Anja Tuulonen

**Affiliations:** 1 Department of Ophthalmology, Oulu University Hospital, Box 2190029, Oulu, Finland; 2 University of Helsinki, Helsinki, Finland; 3 Faculty of Health Sciences, University of Oulu, Oulu, Finland; 4 Tays Eye Centre, Tampere University Hospital, Tampere, Finland

**Keywords:** Glaucoma, Screening, Cost-effectiveness

## Abstract

**Background:**

To describe the rationale and design of the Northern Finland Birth Cohort (NFBC) Eye Study.

**Methods:**

The NFBC Eye Study is a randomised prospective cohort study. The original NFBC study population consists of 12058 subjects born in the region of Lapland and the Province of Oulu. A postal questionnaire covering extensively the medical and socioeconomical background was sent to the 10300 subjects of the NFBC alive and residing in Finland. For the NFBC eye study the subjects were randomised to the screening group (50%) and the control group (50%). The screening protocol includes the following tests: automated and manifest refraction, best corrected visual acuity, central corneal thickness, intraocular pressure, Humphrey 24–2 perimetry, stereoscopic optic nerve head (ONH) and retinal nerve fibre layer (RNFL) photography and imaging with Scanning Laser Ophthalmoscopy (HRT), Scanning Laser Polarimetry (GDx) and Optical Coherence Tomography (OCT).

Two ophthalmologists evaluate the ONH and RNFL photographs and the visual fields independently. All suspected glaucoma cases are re-evaluated by two independent glaucoma experts. HRT, GDx and OCT findings are assessed separately. In the future, both groups (100%) will be examined. The effectiveness and the cost-effectiveness of glaucoma screening will be calculated. The response rate of the questionnaire was 67% (n = 6855) and 871 randomised subjects had undergone the eye screening protocol by the end of April 2013.

**Discussion:**

The trial is designed to address the following questions: what is the best combination of diagnostic tests for detecting glaucoma in an unscreened population, what are the benefits and disadvantages of the screening to the individual and the society and is glaucoma screening both effective and cost-effective. The prevalence, incidence and risk factors of glaucoma and other eye diseases will be evaluated, as well as their impact on quality of life.

## Background

Open angle glaucoma (OAG) is a major cause of avoidable blindness worldwide. Due to its insidious nature and irreversible consequences, OAG is considered to fulfil the criteria for population screening. However, there are no randomised controlled trials that demonstrate the effectiveness of screening for preventing blindness from OAG [1-3].

OAG is the most common type of glaucoma with a prevalence of 1.5-2% in the Caucasian population aged over 50. The worldwide number of OAG patients is estimated to be 61 million and approximately 6.7 million of them are bilaterally blind due to glaucoma [4]. The onset and progression of the chronic optic neuropathy due to OAG is asymptomatic until the late phase of the disease when central vision is also affected. The progression rate of OAG varies, but it has been estimated to take a mean time of 23 years without treatment and 35 years with treatment to progress from mild visual field (VF) damage to at least unilateral blindness [1]. Still up to 5% of the patients with newly diagnosed OAG are blind in at least one eye [5]. A retrospective study in Finland revealed the cumulative incidence of unilateral blindness to be 6% at five years, 9% at 10 years and 15% at 15 years after glaucoma diagnosis. Risk factors for impending blindness were advanced stage of the disease at diagnosis, pseudoexfoliation syndrome, poor compliance and fluctuation of intraocular pressure [6]. As for the risk factors of OAG itself, increasing age, elevated IOP, family history of glaucoma, pseudoexfoliation syndrome, myopia and African origin have been identified so far [7].

To date there is no systematic glaucoma screening in any country and most glaucoma cases are diagnosed by chance. Epidemiological studies have shown that at least half of the glaucoma patients are still undiagnosed [8,9]. An organized screening program could be a cost-effective strategy in older age groups (75–79 years of age) based on a synthetic Markov model comparing regular screening to opportunistic case-finding [2]. However, the results were sensitive to the estimates of several parameters, especially screening cost and specificity of screening tests. In screening, there should be a validated test with good sensitivity and specificity. This has been a major problem in glaucoma screening: there is no “golden standard” for the diagnosis and definition of the OAG is highly subjective depending on the observer and the set of tests. The definition of abnormality is based on the normative data of different diagnostic elements and the combination of them [10]. Furthermore, structural abnormalities in the optic nerve head (ONH) and retinal nerve fiber layer (RNFL) may precede functional defects in the visual fields. In a cross-sectional setting, the mean correlation between the structural and functional tests is only 35% (range 22-59%) [7]. In the past decades diagnostic imaging instruments have been developed to overcome the subjectivity of the evaluations in the diagnostics. Still it is apparent that a single test is insufficient to find the persons with or without glaucoma and the best combination of the tests is unknown. In screening, a reliable protocol for the detection of true glaucoma is more important than early detection.

The NFBC main study was set out to explore the long-term morbidity, disease markers, spectrum of symptoms and psychosocial wellbeing throughout the life span. This unselected, geographically defined population sample consists of individuals born in 1966. The NFBC Eye Study includes three separate entities: the diagnostics of glaucoma, the effect of screening on glaucoma incidence and the cost-effectiveness of screening for glaucoma and other eye diseases. The analysis for the diagnostics is based on both cross-sectional and cohort designs whereas the analysis for the cost-effectiveness is based on randomised controlled design. Hence, the trial seeks to address the following questions: (i) what is the best combination of diagnostic tests for detecting glaucoma in an unscreened population, (ii) what are the benefits and disadvantages of the eye screening to the individual and the society and (iii) is glaucoma screening both effective and cost-effective. Other eye diseases as well as related factors, e.g. retinal perfusion parameters, diabetic retinopathy, early changes related to macular degeneration, presbyopia and vision associated quality of life, are also assessed. Also the relationship between birth weight and growth patterns during early life to eye diseases is evaluated. The purpose of the NFBC Eye Study is to evaluate the diagnostic methods and cost-effectiveness of glaucoma screening as well as other ocular morbidity in middle-aged Caucasian population.

## Methods

### The NFBC main study

**Figure 1:**
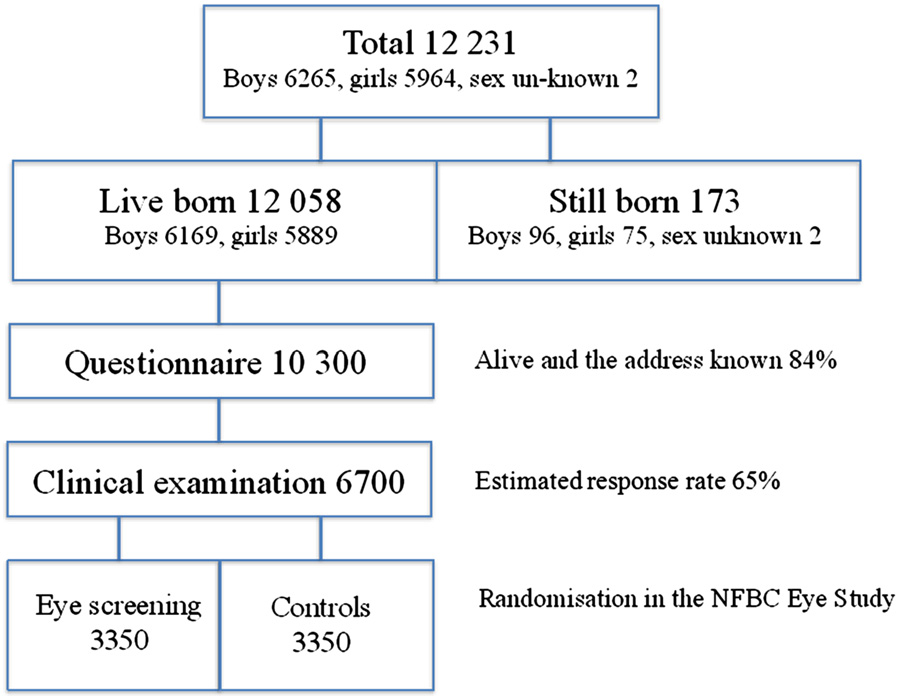
The NFBC Study population.

The original cohort consists of 12058 subjects born in the region of Lapland and the Province of Oulu (96% of the deliveries in these regions). The cohort is comprised of males and females in approximately equal proportions (Figure 1). The mothers of the subjects were recruited to the study on the basis of the expected birth date in 1966. This unique cohort has been followed prospectively since the 24^th^ gestational week. The course of the delivery and neonatal outcome has been confirmed from the patient records. The original data has been supplemented by the information collected with postal questionnaires and clinical examinations carried out at the age of 1, 14 and 31 years. In the previous NFBC analysis during 1997–1998 the response rate to the questionnaire was 75% [11]. Furthermore, data on morbidity, mortality and socioeconomic factors have been collected from national registers and hospital records.

#### Questionnaire and clinical examinations

The postal questionnaire was sent to the 10 300 subjects alive and residing in Finland. The questionnaire is designed to cover extensively the medical and socioeconomical background of the subjects. The ophthalmological portion includes questions on both personal and family history of eye diseases and treatment, including surgery. Information is also gathered on visual acuity, symptoms of presbyopia, refractive error, the use of eyeglasses and contact lenses as well as ocular and vision-related symptoms and quality of life, including the 15D instrument [12]. The entire cohort attends an extensive clinical examination including physiological, cardiorespiratory, orthopedic, dermatological, cognitive and dental status. Comprehensive laboratory tests are also taken.

### The NFBC eye study

The NFBC Eye Study is conducted in co-operation with the main NFBC 1966 Study. The study has received approval from the Ethical Committee of the Northern Ostrobothnia Hospital District. The cohort was randomised to two groups: 50% undergo the eye examination and 50% are not examined. Thus, half of the cohort will be screened and the other half represents the opportunistic case finding model. The subjects randomised to the screening arm with a former diagnosis of glaucoma will undergo the screening protocol in order to evaluate the accuracy of the diagnosis. The pilot study was conducted in 2012 in order to test the study flow. Participants of the pilot study were healthy volunteers from the general population with a mean age of 49 years (n = 80).

### Randomisation

Out of the original cohort, 460 subjects have died and 961 have moved abroad. 10300 subjects with an address in Finland were included in the randomisation. The participants were stratified into three categories based on gender, age and postcode. The subjects were divided into four age categories according to their month of birth: January-March, April-June, July-September and October-December. They were divided into 13 postcode categories roughly following the regional centres of the national mail agency Itella. All in all, there were 104 (2 × 4 × 13) strata for randomisation. Randomisation was performed using Resampling Stats software (Resampling Stats Inc., Arlington, Virginia, USA). The epidemiologist responsible for the randomisation was blinded to all other information than the factors used for stratification.

### Screening

The subjects randomised to the eye screening group (50%) undergo the examinations presented in Table 1. The examinations are performed by experienced study personnel (optometrist, perimetrist and photographer). Each subject is examined only once. An ophthalmologist will not participate in the examination of the subjects but is available for consultation. Up to ten subjects are examined daily, each of them requiring an average time of 65 minutes for eye screening with this comprehensive protocol.

**Table 1:** The eye examinations

**Examination**	**Examiner / instrument**	**Method / protocol**	**Outcome measure**
Autorefraction	Nidek AR-360A		Refraction, near add
Automated visual acuity	Nidek AR-360A		Snellen visual acuity
Subjective refraction	Optometrist		Refraction, near add
Best corrected visual acuity	Optometrist	LogMAR -chart	LogMAR visual acuity
Automated perimetry	Humphrey Field Analyser	Sita standard 24-2	Glaucoma Hemifield Test, Visual Field Index, Mean Deviation, Pattern Standard Deviation
Tonometry	Icare, Goldmann Applanation Tonometer		Intraocular pressure
Stereoscopic ONH photography	Canon CF-60DSi (EOS-1)	Gray-scale	Evaluation for glaucomatous damage
RNFL photography	Canon CF-60DSi (EOS-1)	Grey-scale (495 nm filter)	Evaluation for glaucomatous damage
Fundus photography	Canon CF-60DSi (EOS-1)	Colour and gray-scale	
Optic nerve head OCT	Cirrus 4000	Optic Disc Cube 200 × 200	Rim thickness, Rim area, Cup:disc ratio
Macular OCT	Cirrus 4000	Macular Cube 512 × 128	Macular thickness
Central corneal OCT	Cirrus 4000	Anterior Segment HD Images	Central corneal thickness
Chamber angle OCT	Cirrus 4000	Anterior Segment HD Images	Angle opening distance
Pachymetry	Tomey SP-3000		Central corneal thickness
Scanning laser polarimetry	GDx Pro	ECC	RNFL-I Summary Parameters, Nerve Fiber Indicator
Optic nerve head topography	HRT3		Moorfields Regression Analysis, Glaucoma Probability Score, Stereomeric parameters

By the end of April 2013 the response rate of the questionnaire was 67% (n = 6855) and 871 randomised subjects had undergone the eye screening protocol. The enrolment for the clinical examination is still in progress. Informed consent has been obtained from all subjects attending examinations.

### Diagnostics of glaucoma

#### Visual fields

The visual field examination is performed using the 24–2 Swedish Interactive Threshold Algorithm (SITA) of the Humphrey Field analyser II-*i* (Humphrey Instruments, San Leandro, California). Static white-on-white perimetry (white test points on a white background) limited to the 24 degree central area is considered to be the current standard for detecting glaucomatous visual field defects [7,10,13]. Numerical data e.g. reliability indices, glaucoma hemi-field test (GHT), visual field index (VFI), mean deviation and pattern standard deviation are documented. Evaluation of the overall reliability and suspicion of glaucoma is made by two independent ophthalmologists. Suspicion of early glaucomatous field loss is based on the following three parameters: reduction of sensitivity at the minimum of three clustered points with significance of p < 0.05 and one of them with significance of p < 0.01 on pattern deviation map, glaucoma hemi-field test (GHT) regarded as “borderline” or “outside normal limits” or pattern standard deviation abnormal at p < 0.05 level. The visual fields are considered to be reliable if the rate of false-positives is less than 15% and the rate of fixation losses is less than 20%.

#### Stereoscopic ONH and RNFL imaging

Color and grayscale digital fundus images are obtained with a Canon CF-60DSi Digital Mydriatic Fundus Camera with attached Canon EOS-1Ds MK III SLR Digital Camera (Canon Inc., Tokyo, Japan). The pupils are dilated for imaging. The images are processed with Adobe Photoshop CS (Adobe Systems Inc., San Jose, CA, USA) and Neacapture software (Neagen Ltd., Oulu, Finland). Stereoscopic ONH photography still represents the standard for detecting glaucomatous damage and progression of the optic disc [14]. The separate RNFL photographs are taken with a monochromatic blue interference filter (495 nm) using the camera described above. The technique is a refined digital version of a well-documented method used in the Oulu University Hospital for more than three decades [15,16]. The RNFL photographs are taken and processed by an experienced photographer. The screening evaluations of the RNFL and ONH photographs are performed by two independent general ophthalmologists.

#### Modern imaging technologies: HRT, GDx and OCT

The evaluation of optic nerve head topography is carried out using the Heidelberg Retina Tomograph (Heidelberg Engineering, Heidelberg, Germany; HRT3, image acquisition software version 3.1.2a, Heyex 1.6.2.0). The HRT explores the ONH and the adjacent retinal nerve fibre layer at stepwise progressing depths. The stack of captured images is used to form a three-dimensional topography image. The stereometric optic nerve head parameters (e.g., cup:disc area ratio and cup shape measure), the Moorfield’s regression analysis (MRA) and Glaucoma probability score (GPS) are calculated from the topography image [17-19]. The findings are compared to normative data and evaluated for glaucomatous damage.

Scanning laser polarimetry is carried out with the GDxPRO with Enhanced Corneal Compensation (software version 1.1.0; Carl Zeiss Meditec, Dublin, CA). The RNFL polarizes light making the reflected beam proceed in two focus planes (“birefringence”). The thickness of the RNFL is related to the alteration of the polarized light. The enhanced corneal compensation mode is used to improve signal-to-noise ratio and the quality of the image. It has been shown to improve diagnostic accuracy for glaucomatous damage [20]. The RNFL-I summary parameters (TSNIT, superior and inferior average, inter-eye symmetry, and nerve fibre indicator) are evaluated. There is evidence suggesting that RNFL micro-structures undergo changes in orientation and density before other structural changes become apparent [21,22].

Cirrus HD-OCT 4000 (software version 6.0.0; Carl Zeiss Meditec) is used to obtain spectral domain optical coherence tomography (OCT) images. Anterior Segment HD Images (5 Line Raster) are obtained from the central cornea and limbus to evaluate central corneal thickness and the angle opening distances of the anterior chamber angle. The standard Macular Cube 512 × 128 protocol is used to evaluate macular thickness parameters and the Optic Disc Cube 200 × 200 protocol to assess the peripapillary ONH parameters. The ONH parameters acquired with Cirrus HD-OCT have been shown to be able to discriminate between normal and glaucomatous eyes [23].

### Study flow

In this study, the definition of glaucomatous damage is based on the examinations of the ONH, RNFL and VF and Finnish Evidence Based Guideline '2 out of 3 rule’. When at least two out of three of the examinations are found to be abnormal, glaucoma is likely [7]. IOP is documented but not included in the definition of glaucoma. Epidemiological studies have shown that up to 40% of the patients with glaucomatous damage have normal IOP (normal tension glaucoma) and that all patients with elevated IOP do not develop glaucomatous damage [24,25]. The findings of the HRT, GDx and OCT examinations are analysed separately in order to compare their sensitivity and specificity to Finnish Evidence guideline in the screening of unselected population.

**Figure 2:**
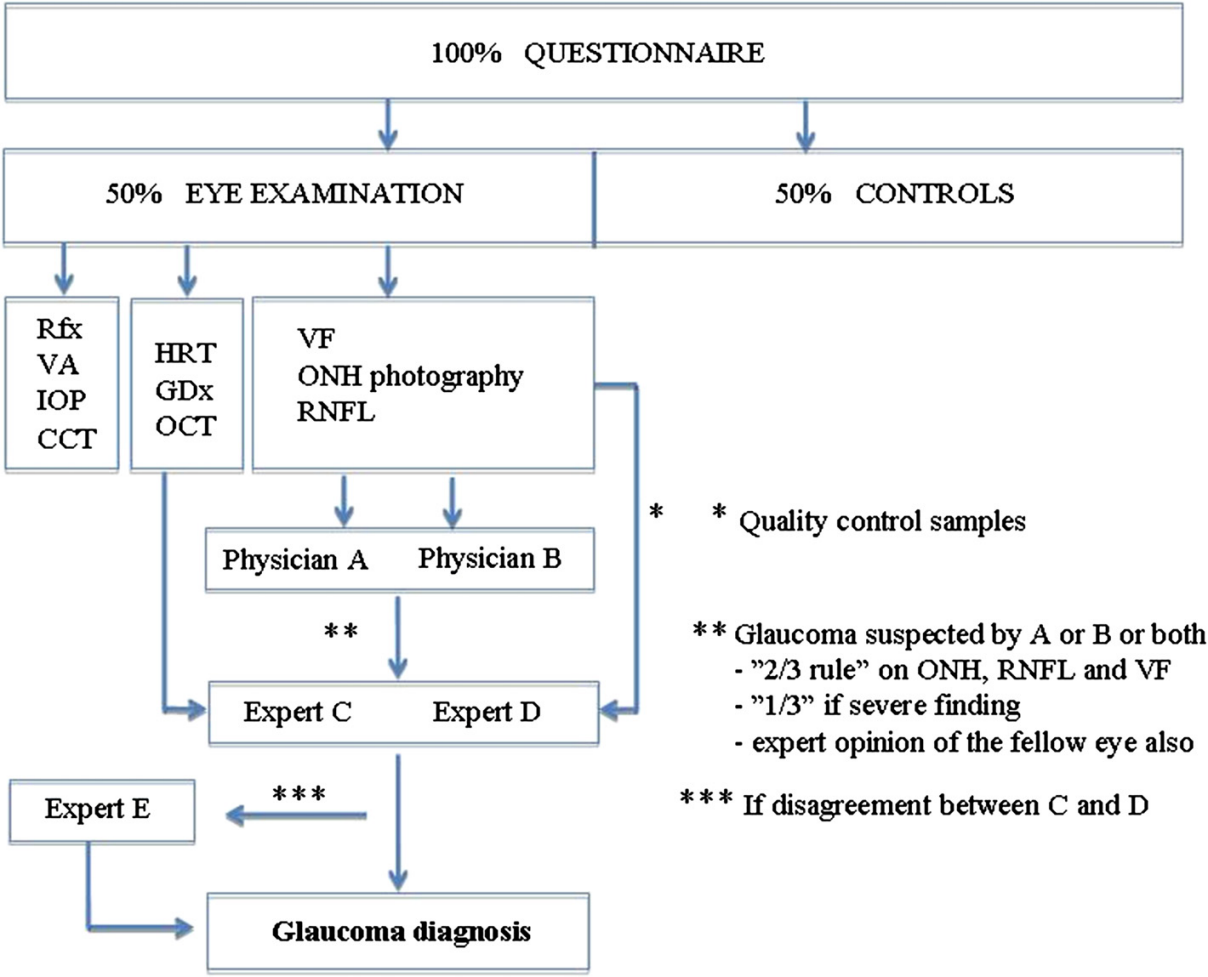
**The study flow.** Glaucoma is suspected by two independent general ophthalmologists (A and B) or based on the findings of imaging technology (HRT, GDx, OCT). The diagnosis is confirmed by glaucoma experts (C, D and E).

Two ophthalmologists (EK and KS) evaluate the ONH and RNFL photographs and visual fields independently. All suspected glaucoma cases (abnormalities in '2 out of 3’ or '1 out of 3’ in case of a severe finding) found by EK or KS or both are re-evaluated by two independent glaucoma experts (ML and PH) to confirm the diagnosis. A third glaucoma expert (ATu) will re-evaluate the findings in case of disagreement between the two glaucoma experts. If glaucomatous damage is identified by the imaging instruments (HRT, GDx, OCT) in subjects not considered glaucoma suspects by the ophthalmologists, they are re-evaluated by the glaucoma experts. A random sample of subjects with normal findings is also re-evaluated by the experts for quality control (Figure 2). All subjects with suspected or diagnosed glaucoma are referred to the hospital eye department for follow-up and consideration for treatment. After 10 years’ time, starting in 2023, the entire cohort (100%) will be examined with a similar protocol. The clinical and cost data of the unscreened arm will be then collected retrospectively from health care records.

### Statistical analyses

IBM SPSS Statistics v. 20 is used for statistical analyses. Continuous data will be analyzed by two-tailed unpaired *t*-test and categorical data by χ^2^ test. Frequencies and standard deviations for the baseline characteristics and the risk ratios for eye morbidity will be calculated. The sensitivity and specificity of the diagnostic tests and inter-observer agreement rate on glaucoma detection will be assessed. Later on, the risk ratios for developing glaucoma and visual disability in the screened and unscreened group are compared. The cost-effectiveness of the screening will be calculated in terms of gained QALY’s and avoided years of visual disability [1,2]. Statistical significance will be reported when p < 0.05.

## Discussion

The NFBC 1966 cohort is a well-documented, structured and lead cohort. It is a large cohort with more than 10 000 subjects in follow-up. It is population-based and very homogenous in respect to many important, potentially confounding, factors. Hence, the results are applicable to populations with a Caucasian ethnic origin.

The cohort is unique as the subjects have been prospectively followed since the 24^th^ gestational week. In addition to the glaucoma screening tests, also examinations suitable for diagnosing other eye diseases are obtained, e.g. macular OCT and fundus photography. The prospective follow-up allows the evaluation of the relationship between birth weight and growth patterns during early life to eye diseases. Risk factors and their value in predicting future glaucoma and other eye abnormalities will also be assessed.

The main challenge for glaucoma research is the lack of the diagnostic reference method, i.e. the golden standard. Any single conventional or modern diagnostic test is not reliable enough to detect a new or progressing glaucoma on its own. To date the diagnosis and determination of stability or instability of glaucoma has been based largely on the subjective expert opinion on structural and functional tests. The opinion between experts still tends to vary: the range of kappa statistic strength of agreement in different cross-sectional studies has been 0.5-0.9 [7]. From this point of view, objective imaging tools are expected to be useful to get a more objective evaluation of the RNFL and ONH. Still, the management decisions should not be based solely on the results of a single technology [26,27]. The visual fields and the imaging results of HRT, GDx and OCT are also subject to fluctuation. Glaucoma is a progressive optic neuropathy and the variability of test results disturb the detection of progression. Progression during follow-up will be used a reference standard for the diagnosis as progression is a part of the definition of glaucoma [28].

Repeated testing has been shown to reduce the variability of test results [29]. However, in a population screening setting, it is not feasible to perform repeated screening tests to all subjects. In the current study, only subjects with glaucomatous findings are referred to hospital for treatment and follow-up. It should be noted that this trial offers meticulous eye examinations for glaucoma screening but not actual treatment and care.

The effectiveness of screening can best be demonstrated by a randomised controlled setting. To date randomised diagnostic studies in glaucoma have been missing [24]. To our knowledge, this study is the first randomised controlled trial on glaucoma screening in an unselected population. Optimal intervention and prevention of the diseases may be possible if the high-risk groups are detected early. It is known that no single test is sufficient to discriminate persons with and without glaucoma but the optimum set and number of diagnostic tests is unknown [1]. Moreover, a single examination of functional damage may not be reliable and the repeated test should be done in case of suspected damage.

In 10 years’ time the entire cohort (both the screened and the controls) will be examined with a similar screening protocol described here. The incidence of glaucoma and other eye diseases in both groups will be evaluated and the effectiveness and cost-effectiveness of glaucoma screening for preventing blindness can be assessed. So far, there has not been compatible evidence for calculating the cost-utility of glaucoma screening [1,2]. A European multi-centre randomised screening trial has been suggested to evaluate cost-effectiveness in preventing glaucoma induced visual impairment. The NFBC eye study is designed to provide the much needed high quality evidence for the evaluation of the effectiveness and cost-effectiveness of glaucoma screening.

## Competing interests

The authors declare that they have no competing interests.

## Authors’ contributions

VS participated in the design of the study, data analysis and writing the manuscript. EK participated in data analysis and writing the manuscript. KS, PH and ML participated in data analysis, the evaluation of glaucomatous damage and revising the manuscript. JK performed the randomisation and participated in the design of the study and writing the manuscript. ATa participated in the design of the study and revising the manuscript. ATu participated in the design of the study, data analysis and writing the manuscript. All authors have approved the final manuscript.

## Pre-publication history

The pre-publication history for this paper can be accessed here:

http://www.biomedcentral.com/1471-2415/13/51/prepub
